# The persistence and complexity of disordered sleep in well-controlled HIV infection

**DOI:** 10.1093/sleep/zsaf104

**Published:** 2025-04-17

**Authors:** Malcolm von Schantz, Karine Scheuermaier

**Affiliations:** Faculty of Health and Life Sciences, Northumbria University, Newcastle-upon-Tyne, UK; Wits Sleep Laboratory, Brain Function Research Group, Department of Physiology, School of Biomedical Sciences, Faculty of Health Sciences, University of the Witwatersrand, Johannesburg, South Africa

Published reports of disordered sleep started to emerge early in the HIV pandemic [1]. It was noted that altered sleep architecture was also observed in men with otherwise asymptomatic HIV infection [2]. At that time, deaths caused by HIV as the underlying cause were still on the rise in the United States and the pandemic was still spreading rapidly and devastatingly across the world, especially Africa. Sleep, during that era, was not high on the priority list for clinical management of people with HIV. Today, more than three decades later, emerging reports, such as the one published in this issue by Punjabi and colleagues [3], suggest that the time has come for this to change.

Advances in the efficacy and global availability of antiretroviral therapy (ART) mean that millions of people worldwide are now living with HIV as a long-term condition with full viral suppression (subject to continued and unrestricted availability of ART and compliance with treatment). This has shifted the main clinical challenge away from preventing morbidity caused by the propagation of the HIV virus itself, and opportunistic infections enabled by it, toward long-term prevention and management of noncommunicable comorbidities. People with HIV suffer an increased burden of the many noncommunicable diseases (NCD) which associated with aging for reasons that are not clearly understood. This includes disordered sleep, which according to a systematic review and meta-analysis, affects 58% of people with HIV [4]. A direct comparison with lifestyle-matched controls concluded that people with HIV had an increased risk of insomnia with an adjusted odds ratio of 5.3 [5]. The profile of the additional NCD burden in people with HIV is not dissimilar to the known direct effects of long-term sleep disturbances, and it has been suggested that disordered sleep may mediate part of the unexplained morbidity burden [6].

Punjabi and colleagues performed home polysomnography (PSG) in 447 participants with HIV and 349 control participants, both recruited from the Multicenter AIDS Cohort study, using a self-applied recorder (NoxA1) [3]. This relatively noninvasive PSG method could reasonably be predicted to minimize first-night effects. Nonetheless, both participants with and without HIV recorded a very low proportion of N3 (4.0 and 3.7%, respectively, *p* = .61), which to us seems likely to be an artifact of either the protocol or the technology (or both). Although the participants with HIV were younger (56 vs. 63, *p* < .001), they had longer sleep latency (20.9 vs. 16.9 min, *p* = .03) and shorter total sleep time (but also shorter time in bed), suggesting poorer sleep health at an earlier age in people with HIV. A striking difference was observed when participants were stratified by sleep-disordered breathing (SDB) as measured by the apnea–hypopnea index (AHI). The groups of people with and without HIV had similar prevalence of moderate to severe SDB (AHI ≥ 15). However, participants with HIV in the highest AHI category (≥30 events/h), and thus with the most severe SDB, had lower sleep efficiency and a higher percentage of N1, as well as fewer transitions from NREM to REM. Thus, severe SDB was observed with the same prevalence in the population of people with HIV (even though they were younger) and with more severe effects on sleep architecture. This suggests an underlying additional burden of sleep vulnerability in people with HIV.

The same authors have previously published a paper from the same cohort reporting mild and moderate SDB to be more common in people with HIV [7]. Their current report suggests that, regardless of whether obstructive sleep apnea (OSA) above the clinical threshold is more common in people with HIV, the effect of higher levels of SDB are more severe. This is an important observation for several reasons. First, there is evidence suggesting that OSA in people with HIV may not follow the generally recognized risk factors (obesity, male sex, age above 50, high neck circumference) [8]. Furthermore, most people living with HIV reside in sub-Saharan Africa, where OSA rates are on the rise whilst the availability of CPAP treatment is very limited [9].

In their discussion, Punjabi and colleagues [3] paint a picture in which the many subjective reports of poor and disrupted sleep in people with HIV, even when well controlled, are being corroborated through objective studies, providing suggestions for potential underlying mechanisms such as immune activation [10] and circadian misalignment [11], in addition to the potential adverse effects of the daily ART regime [12]. This suggests that sleep in people with HIV is already vulnerable, which could be an important part of the reason why the additional challenge of severe SDB results in disproportionate disruption to sleep architecture. These findings highlight the importance of future research aiming towards a greater understanding of sleep vulnerabilities in HIV and appropriate specific interventions.
